# An Adaptive Optimization Method for Acoustic Temperature Measurement Topology Based on Multiple Sub-Objectives

**DOI:** 10.3390/s25061878

**Published:** 2025-03-18

**Authors:** Jialiang Zhu, Xinzhi Zhou, Hailin Wang, Yixiao Chen, Tao Xu, Zhengxi He

**Affiliations:** 1 College of Electronics and Information Engineering, Sichuan University, Chengdu 610065, China; npiczhujialiang@126.com (J.Z.); yixiao_chen@stu.scu.edu.cn (Y.C.); 2 National Key Laboratory of Nuclear Reactor Technology, Nuclear Power Institute of China, Chengdu 610213, China; hailin_lie@163.com (H.W.); xutaonpic@163.com (T.X.); zhengxihe_npic@163.com (Z.H.)

**Keywords:** acoustic tomography, adaptive optimization, asymmetric distribution, temperature field reconstruction, topology, multi-objective

## Abstract

Recent years have seen a surge in study interest in acoustic temperature measurement because of its exceptional non-invasiveness, high precision, and fast response characteristics. Its main benefit is that it may rely on the temperature field reconstruction technique to obtain the entire temperature distribution information, circumventing the limitations of point-type thermometry. Studies have shown that the acoustic wave transducer topology is a key factor affecting the reconstruction effect. In engineering, a simple uniform placement or trial-and-error methods are often used to determine the transducer topology. However, these approaches lack adaptability in complex temperature fields, resulting in poor accuracy and stability. In this paper, based on the previous research on high-precision temperature field reconstruction algorithms, an adaptive optimization method of acoustic temperature measurement topology based on multiple sub-objectives is proposed. The method further improves the reconstruction of asymmetric complex temperature fields by constructing a new optimization variable and a new optimization objective. Comparison experiments with existing optimization methods demonstrate the effectiveness of the new variables and objectives. Additionally, the reconstruction performance of the proposed method is thoroughly evaluated. The results indicate that the method enables adaptive optimization of transducer topology. Moreover, the optimized results exhibit high accuracy and stability in reconstructing complex, asymmetric temperature fields.

## 1. Introduction

Acoustic temperature measurement is a common non-contact technique that measures temperature by using the relationship between the velocity of acoustic waves and the medium temperature. Its benefits include excellent real-time performance, high accuracy, a broad measurement range, and strong adaptability [1,2,3,4,5]. In addition, the method is able to break through the limitations of single-point temperature measurement and reconstruct the complete temperature distribution by laying multiple acoustic wave paths [6,7]. However, it is essential to know, in practical settings, whether the measured area’s temperature distribution typically exhibits complicated and changing properties. For example, in a boiler chamber, the temperature field exhibits different characteristics under various conditions. During normal combustion, it typically shows symmetric single-peak characteristics. When the flame contacts water-cooled wall tubes, it displays asymmetric single-peak skewed characteristics. Under poor combustion conditions, it often shows multi-peak characteristics [8]. In contrast, temperature fields in granaries are more complex and diverse due to the larger number of heat sources and their random locations [9]. Therefore, improving the reconstruction of temperature fields with complex asymmetric characteristics—such as asymmetric heat source locations, peak values, and temperature gradients—is both a challenging and an urgent task.

Our team has previously suggested a high-precision temperature field reconstruction algorithm based on the logarithmic–quadratic radial basis function (RBF) and singular value decomposition (SVD), referred to as LQ-SVD, for the aforementioned difficult measurement scenarios [10]. We have combined simulation and experimentation to verify the algorithm’s efficacy in practical engineering applications.

The acoustic transducers’ topology, however, will significantly affect the distribution of the acoustic paths during the temperature field reconstruction process. This affects the path matrix and its sparsity in the reconstruction algorithm, ultimately influencing the temperature field reconstruction effect [11,12]. Therefore, this paper focuses on optimizing the acoustic temperature measurement topology based on existing high-precision reconstruction algorithms. The goal is to achieve high accuracy and stability in various temperature distributions [13].

At present, there are few studies on the optimization of transducer topology. In engineering, simple and uniform arrangements are commonly used, or trial-and-error methods are employed to find a relatively optimal topology. However, these approaches lack adaptability in complex temperature fields and fail to achieve satisfactory accuracy and stability.

To achieve automatic optimization of transducer topology, Song et al. [14] used the particle swarm optimization (PSO) technique. They positioned eight acoustic wave transducers (i.e., eight optimization variables) around the square measured area, with the goal function being the root mean square reconstruction error of a double-peak temperature field. Simulation results show that the optimized topology performs better for reconstructing double-peak temperature fields. However, it is less effective for other temperature fields and even underperforms compared with a uniform topology.

To enhance the resilience of the optimized topology to various temperature fields, Zhang et al. [13], Yan et al. [15], and Zhang et al. [16] modified the objective function to the average relative reconstruction error of a uniform temperature field. Simulation results demonstrate that the optimized topology significantly improves the reconstruction effect for several typical temperature fields, when compared with the uniform topology, and outperforms the method proposed in [14]. To further improve optimization performance, Zhang et al. [11] reduced the optimization variable to one—the distance between each transducer and its adjacent vertex. They used the average relative reconstruction error of a uniform temperature field and the number of grids crossed by sound paths as the objective function. The transducer positions were then optimized using the beetle antennae search (BAS) algorithm. The results show that the topology obtained by this method outperforms the uniform topology and the optimization results of the methods proposed in [13,14,15,16].

However, all of the above four optimization methods have some problems and shortcomings. Firstly, for the design of optimization variables, Refs. [13,14,15,16] use eight variables, meaning that each transducer position parameter corresponds to a variable. The local optimum is quickly reached by the optimization outcomes, and the optimization efficiency is low. Ref. [11] takes into account the symmetry of the transducer position, and chooses one variable, which is the distance from each transducer to the corresponding vertex of the measured area. Although this approach improves the optimization efficiency, the obtained optimization results all have axial symmetry in both horizontal and vertical directions, resulting in insufficient acoustic coverage. Second, in the design of optimization objectives, Ref. [14] selects the root mean square error of a double-peak temperature field as the objective function. However, this approach is too limited, as the optimization results are not suitable for measurement scenarios beyond double-peak temperature fields. Refs. [11,13,15,16] all consider the universality of the optimization results, and choose a uniform temperature field as the optimization object. However, Refs. [13,15,16] only construct a single objective function based on the average relative error, without taking into account more factors that may affect the reconstruction effect. Ref. [11] constructed a dual-objective function including the mean relative error and the quantity of acoustic paths passing through the grid, which is better optimized than the single-objective function. In this method, the weight coefficients of the two sub-objectives were set to be equal. However, depending on the temperature distribution characteristics of the measured area, different optimization objectives may contribute to the temperature field reconstruction results to different degrees.

To address the limitations of existing optimization methods, this paper focuses on asymmetric complex temperature fields and proposes a new adaptive optimization method for transducer topology based on multiple sub-objectives. Additionally, the method further improves the impact of temperature field reconstruction on the basis of the LQ-SVD algorithm by designing new optimization variables and constructing new objective functions. The simulation results show that the topology obtained by this method has good accuracy and stability in reconstructing four typical asymmetric complex temperature fields.

## 2. Materials and Methods

### 2.1. Basic Theory

#### 2.1.1. Principle of Acoustic Temperature Measurement

Acoustic temperature measurement is based on the principle that medium temperature affects sound speed, allowing temperature distribution to be inversely derived from sound speed distribution. According to the dynamics, thermodynamics, acoustics and other related theoretical knowledge, the function relationship between sound speed c and medium temperature T is as follows [17,18,19]:

(1) $$c=\sqrt{\frac{\gamma R}{m}T}=B\sqrt{T}$$

In Equation (1), γ is the ratio of the specific heat capacity at constant pressure to the specific heat capacity at constant volume for the gas medium; R is the gas constant, with units of J/(mol·K); m is the molar mass of the gas, with units of kg/mol. In fact, γ, R, m can be integrated into a constant, B, after the gas medium component of the measured environment has been identified. The unit of B is $\sqrt{J/(kg\cdot K)}$. When the gas medium is air, B usually takes 20.03 [20]. From Equation (1), as can be observed, the gas temperature is the only factor influencing the acoustic wave’s propagation speed, i.e., the two form a single-valued functional relationship.

**Figure 1:**
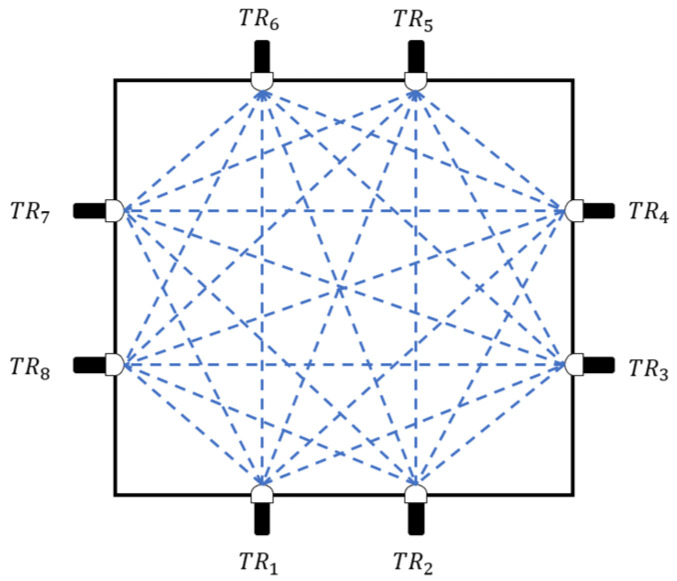
Schematic diagram of multi-path acoustic temperature measurement.

However, most temperature fields in real environments are non-uniform. To address this, scholars have proposed a multi-path temperature distribution measurement scheme [21,22], as illustrated in Figure 1. We uniformly set multiple transducers (there ${TR}_{1}$–${TR}_{8}$) around the temperature field to form many acoustic paths around the region to be monitored (blue dashed line in Figure 1). The distance between two transducers indicates the length of the acoustic path. All other non-identical side transducers receive the matching signal when a particular transducer emits an acoustic signal. For each path, the acoustic time of flight (TOF) is the interval of time between the signals that are sent out and received.

#### 2.1.2. Principle of the LQ-SVD Algorithm

RBF is a mathematical fitting technique that has the advantages of low processing effort and excellent fitting accuracy. It uses linear distance as the single variable, and its linear combination may approximate practically any function [23].

Let f(x,y) be the reciprocal distribution of sound velocity in the observed region. It is assumed that this function is continuous. In order to provide M effective acoustic paths between each acoustic transducer, divide the measured region equally into N sub-temperature zones. For every path, the theoretical value of TOF $t_{k}$ can be written as follows [24]:

(2) $$t_{k}=\int f(x,y)dl_{k}$$

where $l_{k}$ is the *k*th effective acoustic propagation path (k=1,2…M). f(x,y) can be expressed as a linear combination of RBFs at the geometric centroid of the N sub-temperature zones, as follows [25]:

(3) $$f\left(x,y\right)=\sum_{i=1}^{n}\xi_{i}\varphi_{i}\left(x,y\right)$$

where $\xi_{i}$ is the coefficient that needs to be calculated, which characterizes the distribution of $f\left(x,y\right)$. The RBF of the geometrical central point of the ith sub-temperature zone is shown by the symbol $\varphi_{i}\left(x,y\right)$ (i=1,2…N).

This paper’s reconstruction of the temperature field is based on the LQ RBF, which has the following expression in the two-dimensional plane:

(4) $$\varphi_{i}\left(x,y\right)=\ln\left[\frac{\delta^{2}}{{\left(x-x_{i}\right)}^{2}+{\left(y-y_{i}\right)}^{2}+\delta^{2}}\right]$$

where $\left(x_{i},y_{i}\right)$ represents the coordinates of the geometrical center point of the ith sub-temperature zone, or the center of the LQ RBF. Numerical experiments can be used to determine the shape parameter δ.

Associative Equations (2) and (3) can be obtained as follows:

(5) $$t_{k}=\sum_{i=1}^{n}\xi_{i}\varphi_{i}\left(x,y\right)dl_{k}=\sum_{i=1}^{n}h_{ik}\xi_{i}$$

where $h_{ik}=\int \varphi_{i}\left(x,y\right)dl_{k}$. Then, the matrix form can be used to rewrite Equation (5):

(6) t=Hξ

where $t=[t_{1},t_{2},\ldots ,t_{M}{]}^{T},\;\;H=(h_{ij}{)}_{i=1,2,\ldots ,M;j=1,2,\ldots ,N},$ $\xi =[\xi_{1},\xi_{2},\ldots ,\xi_{N}{]}^{T}$.

Equation (6) makes clear that solving this model is really solving an inverse issue, and that matrix inverse problems may be successfully handled by using singular value decomposition (SVD) [26,27].

Singular value decomposition may be used to obtain the inverse matrix $H^{+}$ for the matrix H. Consequently, the weight matrix ξ in Equation (6) can be represented as follows:

(7) $$\xi =H^{+}t$$

By substituting the derived weights ξ into Equation (3), f(x,y) can be obtained. Then, using the functional relationship between temperature and sound velocity, the temperature distribution function T(x,y) in Equation (8) can be derived. This enables high-precision reconstruction of the temperature field.

(8) $$T\left(x,y\right)=\frac{1}{B^{2}f^{2}(x,y)}$$

### 2.2. Topology Optimization Method for Acoustic Transducers

#### 2.2.1. Design of Optimization Variable

**Figure 2:**
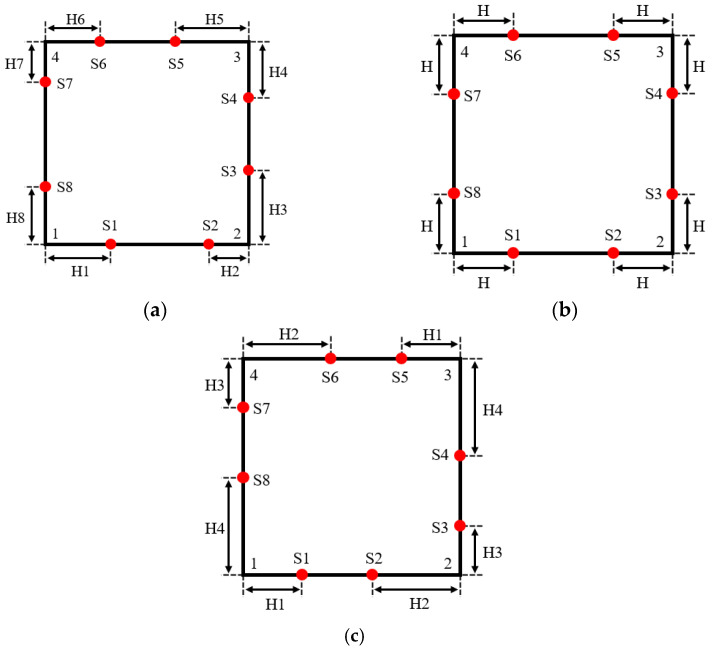
Design scheme for optimization variables. (**a**) Eight optimization variables. (**b**) One optimization variable. (**c**) Four optimization variables.

In order to facilitate the subsequent comparative experiments, 8 acoustic transducers (red dots) are also used in this paper to measure the temperature distribution in the square area. Figure 2 shows the schemes for 8 optimization variables [13,14,15,16], 1 optimization variable [11] and 4 optimization variables (proposed in this paper).

As shown in the figure, for the design scheme of this paper, the eight acoustic transducers are in a centrosymmetric layout. Among these, the distances from S1 to vertex 1 and S5 to vertex 3 are equal as H1; the distances from S2 to vertex 2 and S6 to vertex 4 are equal as H2; the distances from S3 to vertex 2 and S7 to vertex 4 are equal as H3; and the distances from S4 to vertex 3 and S8 to vertex 1 are equal as H4. Therefore, the acoustic measurement topology of this temperature field is determined by the four parameters, namely, H1, H2, H3 and H4, i.e., there are a total of four optimization variables.

Compared with 8 optimization variables, this scheme can not only improve the optimization efficiency, but also more easily obtain the global optimal solution. Compared with using 1 optimization variable, this scheme maintains transducer layout symmetry, simplifying engineering applications, while also improving acoustic path coverage under the same sub-temperature zone division method. Additionally, the optimization results in [15], based on 8 variables, exhibit an approximately centrosymmetric distribution. This further supports the rationality of the 4-variable scheme proposed in this paper.

#### 2.2.2. Design of Optimization Objective

Aiming at the deficiencies of the existing topology optimization methods, this paper proposes a topology optimization method based on multiple sub-objectives. This scheme includes four sub-objectives, namely, acoustic path coverage, acoustic path orthogonality, matrix condition number, and root mean square error. Each sub-objective is described in the following.

(1) Acoustic path coverage

In acoustic temperature measurement, extreme path distributions can leave many sub-temperature zones without acoustic path coverage. This is the main cause of instability and low precision in the inversion problem. To some extent, the issue can be resolved by increasing the quantity of acoustic transducers, the cost factor must be considered in engineering applications. To address this issue with a limited number of transducers, the topology should be designed to maximize the number of sub-temperature zones each acoustic path traverses, thereby extending acoustic path coverage.

Therefore, acoustic path coverage is designated as the first sub-objective (sub-objective 1) in our transducer topology optimization scheme. This is defined as the average number of acoustic paths passing through each sub-temperature zone, expressed as follows:

(9) $$E_{1}=\frac{1}{N}\sum_{i=1}^{N}\rho_{i}$$

where N is the sub-temperature zones’ total number in the measured region, and $\rho_{i}$ is the number of acoustic paths passing through the ith sub-temperature zone. The larger $E_{1}$ is, the more adequately the measured region is sampled, and the better the reconstruction of the temperature field is.

(2) Acoustic path orthogonality

Extreme path distributions not only leave many sub-temperature zones without acoustic path coverage but also cause different acoustic paths to traverse the same or similar sub-temperature zones. This results in redundant temperature information from each acoustic path, reducing the utilization of acoustic path data. Moreover, the high similarity between acoustic paths can cause linear correlations among certain rows in the path matrix H, compromising the stability and accuracy of the inversion problem’s solution. To avoid these issues, the transducer topology should be designed to ensure each acoustic path traverses as many different sub-temperature regions as possible, ensuring the independence of information measured by each path.

Therefore, the second sub-objective (sub-objective 2) in our transducer topology optimization scheme is acoustic path orthogonality. This is defined as the average maximum sine value of the angle between every two acoustic paths within each sub-temperature zone, expressed as follows:

(10) $$E_{2}=\frac{1}{N}\sum_{i=1}^{N}O_{i}=\frac{1}{N}\sum_{i=1}^{N}\max(\sin(\theta_{i}))$$

where N represents all of the observed area’s sub-temperature zones, $O_{i}$ is the acoustic path orthogonality in the ith sub-temperature zone, and $\theta_{i}$ is the angle between the acoustic paths in the ith sub-temperature zone. The larger $E_{2}$ is, the more independent information each acoustic path contains, and the more accurate the temperature field reconstruction will be.

(3) Matrix condition number

During temperature field reconstruction, the path matrix H often exhibits ill-conditioned properties. The degree of ill-conditioning directly affects the reliability of the inversion results.

The matrix condition number, which quantifies the degree of ill-conditioning, is a key metric for assessing the well-posedness of the problem. The condition number of matrix A is determined by multiplying its norm by A and its norm by $A^{-1}$, with the resulting equation being as follows:

(11) $$cond\left(A\right)=\left\|A\right\|\cdot \left\|A^{-1}\right\|$$

The condition number for a system of linear equations Ax=b indicates how sensitive the system’s solution is to b’s uncertainty. A higher condition number indicates greater ill-conditioning, leading to increased sensitivity to measurement perturbations and reduced stability in the numerical solution of the system. During reconstruction, the transducer topology should be designed to minimize the condition number of H in Equation (6), ensuring the stability of the inversion results.

Consequently, the third sub-objective is determined to be the path matrix condition number (referred to as sub-objective 3) of the new optimization scheme of the transducer topology, which is defined as the path matrix’s spectral condition number using the following expression:

(12) $$E_{3}={\left\|H\right\|}_{2}\cdot {\left\|H^{-1}\right\|}_{2}=\frac{\delta_{max}(H)}{\delta_{min}(H)}$$

where ${\left\|H\right\|}_{2}$ and ${\left\|H^{-1}\right\|}_{2}$ are the spectral norms of matrix H and its inverse matrix, respectively. The maximum and minimum singular values of matrix H are denoted by $\delta_{max}(H)$ and $\delta_{min}(H)$, respectively. The inversion’s matrix sickness degree decreases with decreasing $E_{3}$, which also improves the stability of the temperature field reconstruction results.

(4) Root mean square error of the reconstructed uniform temperature field

As previously mentioned, Refs. [11,13,14,15,16] reasonably choose the uniform temperature field as the optimization object. This is because acoustic wave paths in a uniform temperature field sample the measured region more uniformly, and the optimization results are theoretically more generalizable to temperature fields with any shape feature. Thus, one of the crucial indices for assessing the topology’s optimization impact is the reconstruction error of the uniform temperature field. Refs. [11,13,14,15,16] quantify the reconstruction effect of the uniform temperature field using the mean relative error. While this metric partially reflects the reliability of the reconstruction results, it does not capture the magnitude of individual errors. In contrast, the root mean square error is highly sensitive to extreme error values, providing a more accurate representation of the overall reconstruction precision.

Therefore, the fourth sub-objective (sub-objective 4) in our transducer topology optimization scheme is the root mean square error of the reconstructed uniform temperature field, expressed as follows:

(13) $$E_{4}=\frac{\sqrt{\frac{1}{n}\sum_{i=1}^{n}{\left({TR}_{i}-TM\right)}^{2}}}{TM}\times 100\%$$

where n is the number of the calculation points in the measured area and TM is the temperature value of the uniform temperature field model. In the reconstructed temperature field, ${TR}_{i}$ represents the temperature value at the ith calculation point. The smaller $E_{4}$ is, the higher the reconstruction accuracy under uniform sampling conditions and the better the generalization in different temperature fields.

In summary, the transducer topology can be optimized by maximizing $E_{1}$ and $E_{2}$, and minimizing $E_{3}$ and $E_{4}$. In this paper, the objective function E of the above multi-objective optimization problem is constructed using the linear weighted summation method [28,29] as follows:

(14) $$E=\omega_{1}E_{1}+\omega_{2}E_{2}-\omega_{3}E_{3}-\omega_{4}E_{4}$$

where $\omega_{1}$, $\omega_{2}$, $\omega_{3}$ and $\omega_{4}$ are the weighting coefficients, which describe how much each sub-objective contributes to the optimization outcomes. The optimization of the transducers’ topology can be accomplished by maximizing E, thus further improving the temperature field reconstruction results’ accuracy and stability.

In this paper, the complex correlation method is used to calculate the weight coefficients in Equation (14) [30]. This method’s main idea is to calculate the weights according to how strongly the sub-objectives’ complicated correlations are collinear. If the complex correlation between a sub-objective and other sub-objectives is strong, which indicates that there is a large overlap of information, then the sub-objective is given a smaller weight. Conversely, if the complex correlation between a sub-objective and other sub-objectives is weak, indicating that the sub-objective carries a larger amount of information, it is given a larger weight. The following is a description of the method’s specific implementation methodology.

At first, a certain quantity of topological samples are acquired through the random assignment of values to the optimization variables. Each sample has its $E_{1}$−$E_{4}$ computed to create a sub-objective dataset. The dataset is normalized to have a mean of 0 and a variance of 1, addressing potential significant variations in the order of magnitude across sub-objectives. This helps to lessen the impact of outliers on the findings.

Then, the complex correlation between each sub-objective and other sub-objectives is calculated. The complex correlation is measured by the complex correlation coefficient [31], which quantifies the linear relationship between one variable and several others. A higher value indicates a stronger linear correlation. Taking sub-objective 1 as an example, the process of calculating its complex correlation coefficient is as follows.

A multiple linear regression is fitted to sub-objective 1 using the other three sub-objectives, the result can then be expressed as follows:

(15) $$E_{1}^{\prime }=\alpha_{0}+\alpha_{1}E_{2}+\alpha_{2}E_{3}+\alpha_{3}E_{4}$$

where $E_{1}^{\prime }$ is the fitted value and $\alpha_{0}-\alpha_{3}$ are the fitting coefficients obtained by the least squares method. The Pearson correlation coefficient between $E_{1}$ and $E_{1}^{\prime }$ was calculated and expressed as Equation (16), which is the complex correlation coefficient of $E_{1}$.

(16) $$R_{1}=\frac{cov(E_{1},E_{1}^{\prime })}{\sigma_{1}\sigma_{2}}=\frac{\sum \left(E_{1}-\bar{E_{1}}\right){(E}_{1}-\bar{E_{1}^{\prime }})}{\sqrt{\sum {\left(E_{1}-\bar{E_{1}}\right)}^{2}}\sqrt{\sum {\left(E_{1}-\bar{E_{1}^{\prime }}\right)}^{2}}}$$

where $cov(E_{1},$ $E_{1}^{\prime })$ is the covariance of $E_{1}$ and $E_{1}^{\prime }$; $\sigma_{1}$ and $\sigma_{1}^{\prime }$ are the variances of $E_{1}$ and $E_{1}^{\prime }$, respectively; and $\bar{E_{1}}$ and $\bar{E_{1}^{\prime }}$ are the means of $E_{1}$ and $E_{1}^{\prime }$, respectively.

Using the same method, the complex correlation coefficients for other sub-objectives can be calculated. The normalized inverse of these coefficients is then used as the weight for each sub-objective, as follows:

(17) $$\omega_{i}=\frac{\frac{1}{R_{i}}}{\frac{1}{R_{1}}+\frac{1}{R_{2}}+\frac{1}{R_{3}}+\frac{1}{R_{4}}}$$

where $\omega_{i}$ is the weight coefficient of the ith sub-objective (i=1,2,3,4) and $R_{2},R_{3}$ and $R_{4}$ are the complex correlation coefficients of sub-objective 2, sub-objective 3 and sub-objective 4, respectively.

#### 2.2.3. Optimization Process

This research uses the particle swarm optimization (PSO) algorithm, which is based on the optimization objectives and variables mentioned above [32,33]. This algorithm is easy to implement and has a fast convergence and high accuracy for automatic optimization of the transducer topology.

Each particle in the PSO method has two attributes: location and velocity, in the D-dimensional search space. The fitness value, also known as the objective function value, assesses the position’s quality as a potential solution to the issue that has to be optimized. Initially, every particle looks for the best solution (pbest) on its own in the search space and marks it as the best individual solution at that moment. The best individual optimum solution is then chosen as the current global optimal solution (gbest) of the swarm after it has been shared with the other particles in the swarm. At the end of each iteration, each particle updates its position and velocity based on its personal best solution and the global best solution of the entire swarm.

When the number of iterations is k, the updating equations for the nth dimensional velocity $v_{mn}^{k}$ and the *n*th dimensional position $x_{mn}^{k}$ (n=1,2,…,D) of the mth particle are shown in Equations (18) and (19), respectively.

(18) $$v_{mn}^{k}=\omega v_{mn}^{k-1}+c_{1}\;r_{1}\;\left(pbest-x_{mn}^{k}\;\right)+c_{2}\;r_{2}\;(gbest-x_{mn}^{k-1})$$

(19) $$x_{mn}^{k}=x_{mn}^{k-1}+v_{mn}^{k}$$

where ω is the inertia factor, which indicates the degree of inheritance to the current velocity. In contrast, the local optimization ability is strong and the global optimization ability is weak when its value is little. This is because the global optimization ability is stronger when it is large. The individual learning component, denoted by $c_{1}$, represents the weight assigned to the portion of the particle’s subsequent action that is generated from its own experience. The group learning factor, $c_{2}$, represents the weight of the portion of the particle’s next action that is derived from other particles’ experiences. Random numbers with uniform distribution between 0 and 1 are denoted by $r_{1}$ and $r_{2}$, respectively, which are used to increase the randomness of the search. After each round of parameter updates, a judgment will be made. If the termination conditions are not met, the next round of parameter update will be started to continue the optimization search. Instead, if the search termination conditions are met, the search will be ended, and the global optimum solution is the particle’s current location with the highest fitness.

**Figure 3:**
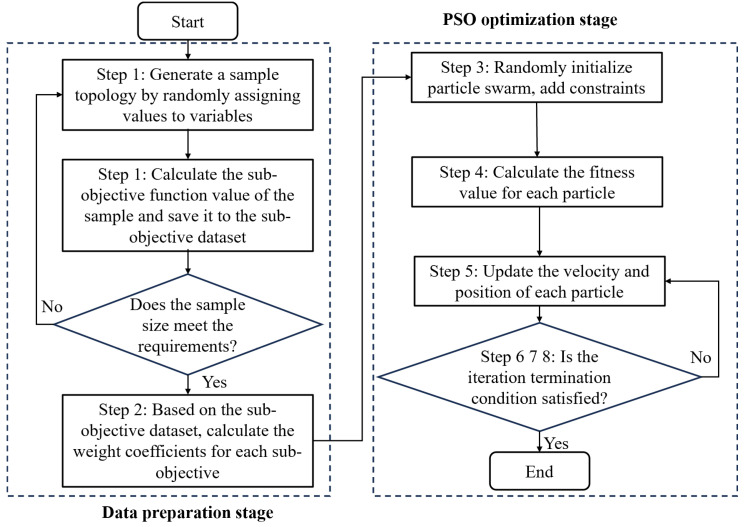
Flow of acoustic temperature measurement topology optimization based on multiple sub-objectives.

Based on the aforementioned theory, Figure 3 depicts the whole flow of the optimization approach suggested in this research, with the following phases being specifically implemented.

Step 1: Generate a predetermined number of topological samples by randomly assigning values to the four optimization variables. Determine the sub-objectives’ values ($E_{1}$−$E_{4}$) for every sample, then enter the results into the sub-objective dataset.

Step 2: Based on the sub-objective dataset, derive the corresponding weight coefficients ($\omega_{1}$−$\omega_{4}$) by calculating the complex correlation coefficients ($R_{1}$−$R_{4}$) of each sub-objective.

Step 3: Set the number of particles, initialize their velocities and positions, and apply constraints (boundary values for velocities and positions) to ensure the transducers remain on the boundary of the measured area without overlapping.

Step 4: Determine each particle’s adaption (E) value individually. Each particle’s starting location is called its individual best position (pbest), and the particle with the highest E among them is called its overall best position (gbest).

Step 5: Update each particle’s position and velocity in accordance with Equations (18) and (19), respectively, and calculate the E corresponding to the current position of each particle in turn.

Step 6: Compare the E-value of the current position of each particle with the E-value corresponding to its pbest, and if the E-value of the current position is greater, replace gbest with the current position.

Step 7: Compare the E-value of the current position of each particle with the E-value corresponding to gbest, and if the E-value of the current position is greater, replace gbest with the current position.

Step 8: Determine whether the iteration termination condition is satisfied, if not, return to step 5 to continue the search. If it is satisfied, end the optimization, and the current global best position gbest is the global optimal solution.

## 3. Experimental Results and Analyses

### 3.1. Experimental Condition

This paper validates the topology optimization method using four typical two-dimensional asymmetric temperature fields: single-peak (${TM}_{1}$), double-peaks (${TM}_{2}$), three-peaks (${TM}_{3}$), and four-peaks (${TM}_{4}$). Their functional expressions are provided in Equations (20)–(23). The four temperature fields are in units of K.

(20) $${TM}_{1}(x,y)=\frac{5000}{0.5\left[{\left(x-1.8\right)}^{2}+{\left(y-2.2\right)}^{2}\right]+10}$$

(21) $${TM}_{2}\left(x,y\right)=\frac{4500}{0.5\left[{\left(x-2.5\right)}^{2}+{\left(y-2\right)}^{2}\right]+10}+\frac{1800}{0.5\left[{\left(x-5.8\right)}^{2}+{\left(y-6\right)}^{2}\right]+5}$$

(22) $${TM}_{3}\left(x,y\right)=\frac{1100}{0.5\left[{\left(x-1.6\right)}^{2}+{\left(y-5.8\right)}^{2}\right]+3}+\;\frac{1000}{0.5\left[{\left(x-4.3\right)}^{2}+{\left(y-1.7\right)}^{2}\right]+3}+\frac{1300}{0.5\left[{\left(x-6.3\right)}^{2}+{\left(y-5.2\right)}^{2}\right]+3}$$

(23) $${TM}_{4}\left(x,y\right)=\frac{850}{0.5\left[{\left(x-2\right)}^{2}+{\left(y-2\right)}^{2}\right]+2}+\;\frac{700}{0.5\left[{\left(x-2\right)}^{2}+{\left(y-6\right)}^{2}\right]+2}+\frac{750}{0.5\left[{\left(x-6\right)}^{2}+{\left(y-6\right)}^{2}\right]+2}+\frac{650}{0.5\left[{\left(x-6\right)}^{2}+{\left(y-2\right)}^{2}\right]+2}$$

**Figure 4:**
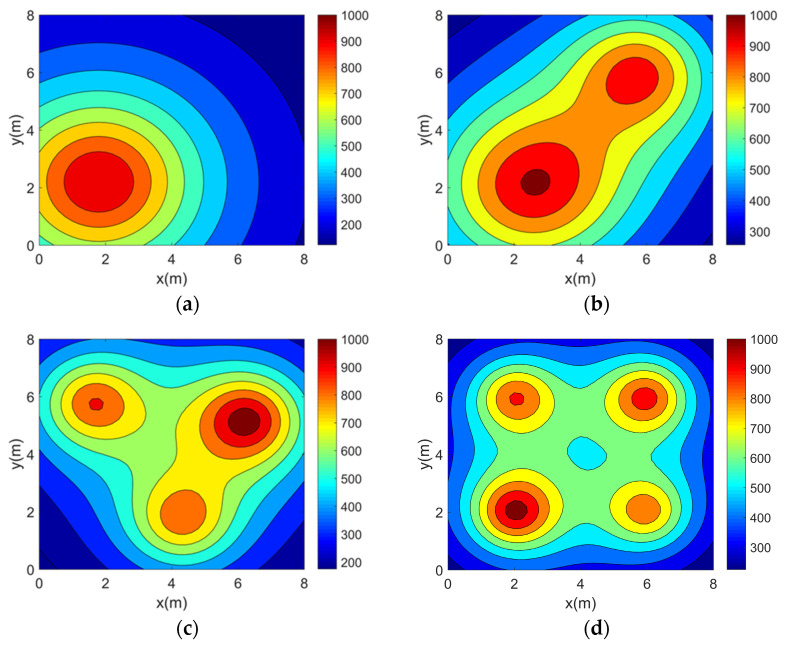
Two-dimensional asymmetric temperature field models. (**a**) Single-peak temperature field model. (**b**) Double-peak temperature field model. (**c**) Three-peak temperature field model. (**d**) Four-peak temperature field model.

In order to visualize the above four 2D temperature field models, their temperature distributions were plotted separately using MATLAB (version 2016b), as shown in Figure 4.

The reconstruction performance is evaluated using three metrics: the maximum relative error ($E_{remax}$), the average relative error ($E_{remean}$), and the root mean square error ($E_{rms}$). These are expressed as follows:

(24) $$E_{remax}=max\left|\frac{{TR}_{i}-{TM}_{i}}{{TR}_{i}}\right|\times 100\%$$

(25) $$E_{remean}=\frac{1}{n}\sum_{i=1}^{n}\left|\frac{{TR}_{i}-{TM}_{i}}{{TR}_{i}}\right|\times 100\%$$

(26) $$E_{rms}=\frac{\sqrt{\frac{1}{n}\sum_{i=1}^{n}{\left({TR}_{i}-{TM}_{i}\right)}^{2}}}{{TM}_{mean}}$$

where n is the number of measurement points for temperature in the area under consideration. In the temperature field model, the temperature value at the ith calculation point is denoted by ${TM}_{i}$. In the reconstructed temperature field, ${TR}_{i}$ represents the temperature value at the ith calculation point. The temperature field model’s average temperature is represented by the variable ${TM}_{mean}$.

In the topology optimization experiments in this paper, the measurement area size is set to 8 m × 8 m, which is uniformly divided into 10 × 10 = 100 sub-temperature zones. As the initial condition, eight acoustic transducers are uniformly placed around the measurement area to form a total of 24 effective acoustic paths, as shown in Figure 1. As for the parameter settings, the number of topology samples in step 1 is 2000, the shape parameter of the LQ-SVD reconstruction algorithm is 2, the particles’ number of the PSO algorithm is 50, the inertia weight is 0.8, the number of iterations is 100, and the group learning factor and the self-learning factor are both 2.

### 3.2. Effectiveness Verification of the Optimization Method

In this section, validation experiments on the effectiveness of the optimization method (including the optimization objective and optimization variable) will be carried out separately based on the optimization process of Section 2.2.3. In order to facilitate the later description, the optimization method in [14] is referred to as Method 1, and its optimization objective is referred to as Objective 1. The optimization methods in [13,15,16] are referred to as Method 2, and their optimization objectives are referred to as Objective 2. The optimization method in [11] is referred to as Method 3, and its optimization objective is referred to as Objective 3. The optimization method in this paper is referred to as Method 4, and its optimization objective is referred to as Objective 4. Additionally, the optimization variables are defined as follows: Variable 1 for Method 1 and Method 2, Variable 2 for Method 3, and Variable 3 for Method 4.

(1) Effectiveness validation of optimization objective

The effectiveness verification of the optimization objective is carried out first. Based on the experimental conditions in Section 3.1, the weights for the four sub-objectives in Objective 4 are determined as $\omega_{1}$ = 0.3193, $\omega_{2}$ = 0.1572, $\omega_{3}$ = 0.1669, and $\omega_{4}$ = 0.3566. The objective function E is expressed as follows:

(27) $$E=0.3193E_{1}+0.1572E_{2}-0.1669E_{3}-0.3566E_{4}$$

The objective function (Objective 4) constructed in this paper assigns greater weight to the root mean square error of the reconstructed uniform temperature field and acoustic path coverage, while reducing the influence of the path matrix condition number and acoustic path orthogonality on optimization performance. To validate the effectiveness of Objective 4—measuring the correlation between reconstruction errors for different temperature fields—this section compares it with Objective 1, Objective 2, and Objective 3. The Pearson correlation coefficients between the above four optimization objectives and the four temperature field reconstruction errors were computed under 100 random topologies, as shown in Table 1, Table 2 and Table 3.

Analysis shows that Objective 1 has an extremely weak correlation with the reconstruction errors of the single-peak, three-peak, and four-peak temperature fields. Although its correlation with the double-peak field is relatively higher (supporting the findings in [14]), it remains weak, indicating that Objective 1 cannot effectively characterize topology reconstruction performance. Objective 2 shows an extremely weak correlation with the reconstruction errors of all temperature fields, with some coefficients (except for the single-peak field) being negative. This suggests that using Objective 2 for optimization may not only fail to improve reconstruction accuracy but could even worsen the results. Compared with Objective 1 and Objective 2, Objective 3 shows significantly stronger correlations with reconstruction errors: weak for the single-peak field, moderate for the double-peak field, and strong for the three-peak and four-peak fields. This indicates that Objective 3 can partially characterize topology reconstruction performance. Notably, Objective 4 exhibits stronger correlations with reconstruction errors than the other three objectives: moderate for the single-peak field and strong for the double-peak, three-peak, and four-peak fields. In summary, Objective 4 better characterizes topology reconstruction performance and is more effective for improving temperature field reconstruction.

(2) Effectiveness validation of optimization variable

In order to verify the validity of Variable 3, it is compared with Variable 1 and Variable 2 in this section. The experimental scheme involves setting the optimization variables as Variable 1, Variable 2, and Variable 3 within Method 4. The resulting topologies are then used to reconstruct the four temperature fields. The error data of the reconstruction results (obtained on the basis of 41 × 41 = 1681 temperature calculation points) are shown in Table 4, Table 5 and Table 6.

Analysis of the error data shows that Variable 3 minimizes all error indicators except $E_{remax}$ for the double-peak and four-peak temperature fields. For Variable 3, the average $E_{remax}$ across the four temperature fields is below 7%, 1.0246% and 1.7299% lower than Variable 1 and Variable 2, respectively. The average $E_{remean}$ is below 1%, 0.5865% and 0.0620% lower than Variable 1 and Variable 2, respectively. The average $E_{rms}$ is below 1.5%, 0.9762% and 0.0620% lower than Variable 1 and Variable 2, respectively. In summary, Variable 3 outperforms Variable 1 and Variable 2 in reconstructing all four temperature fields, making it a more effective optimization variable for improving reconstruction results.

(3) Optimization results and performance evaluation

**Figure 5:**
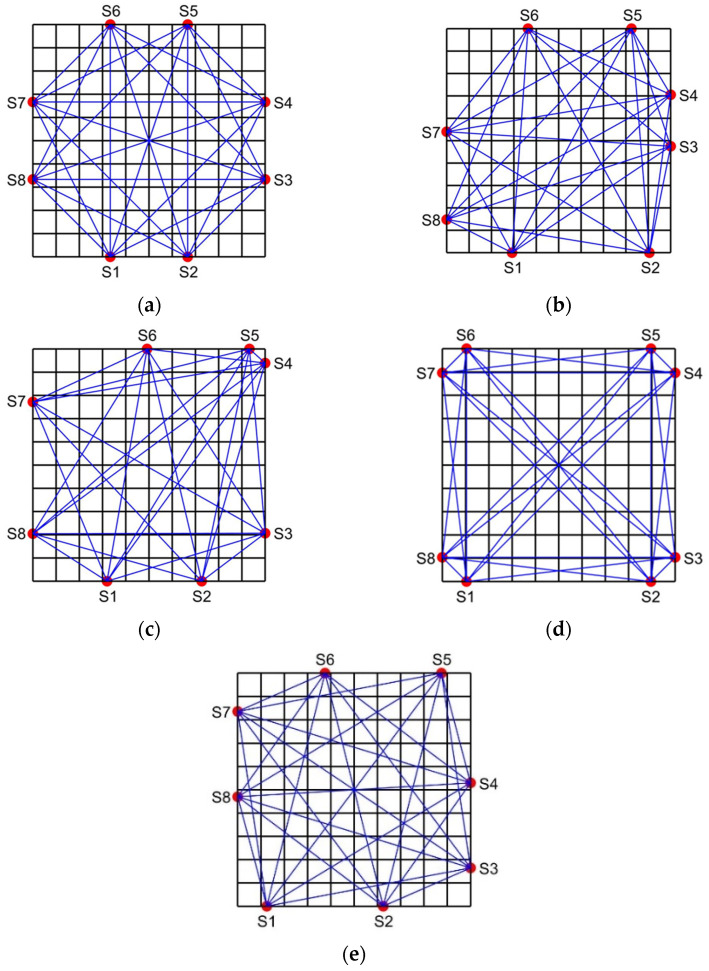
Five topologies and acoustic path distribution. (**a**) Topology 1. (**b**) Topology 2. (**c**) Topology 3. (**d**) Topology 4. (**e**) Topology 5.

The transducer’s topology, which was achieved by optimization using Methods 1, 2, 3, and 4, is depicted in Figure 5, along with the conventional trisection uniform topology. In this paper, we conducted approximately 50 optimization experiments on all optimization methods and found that the experimental results of all optimization methods were relatively stable. Therefore, only one representative experimental result is presented in this paper.

Hereafter, the topologies are labeled as follows: Topology 1 for the trisection uniform topology, Topology 2 for the topology optimized by Method 1, Topology 3 for the topology optimized by Method 2, Topology 4 for the topology optimized by Method 3, and Topology 5 for the topology optimized by Method 4.

Topology 1 exhibits good symmetry and relatively uniform acoustic path distribution. However, some sub-temperature zones near the four vertices of the measured region lack acoustic path coverage, leading to increased reconstruction errors at the edges. Topology 2 and Topology 3 lack symmetry. In Topology 2, S6 and S7 are far apart, resulting in sparse acoustic paths in the upper left corner and insufficient temperature information, compromising reconstruction accuracy. In Topology 3, S4 and S5 are too close, causing dense acoustic paths in the upper right corner and sparse coverage elsewhere, making it difficult to ensure universality across different temperature fields. Topology 4 maintains axial symmetry, but the transducers are too close to the vertices, leading to a nonuniform acoustic path distribution. Many paths traverse the edges and diagonals, while other areas have sparse coverage, resulting in uneven reconstruction accuracy. Additionally, some sub-temperature zones are crossed by only one acoustic path, reducing path quality. In contrast, Topology 5 demonstrates central symmetry and a more uniform acoustic path distribution, avoiding extreme density or sparsity. Most sub-temperature zones are crossed by more than two acoustic paths, ensuring high path quality.

In summary, the optimized transducer topology proposed in this paper provides more uniform acoustic path distribution, enabling higher-quality coverage of the measured region and supporting high-precision reconstruction of various temperature fields.

A. Accuracy experiment of optimization results

**Figure 6:**
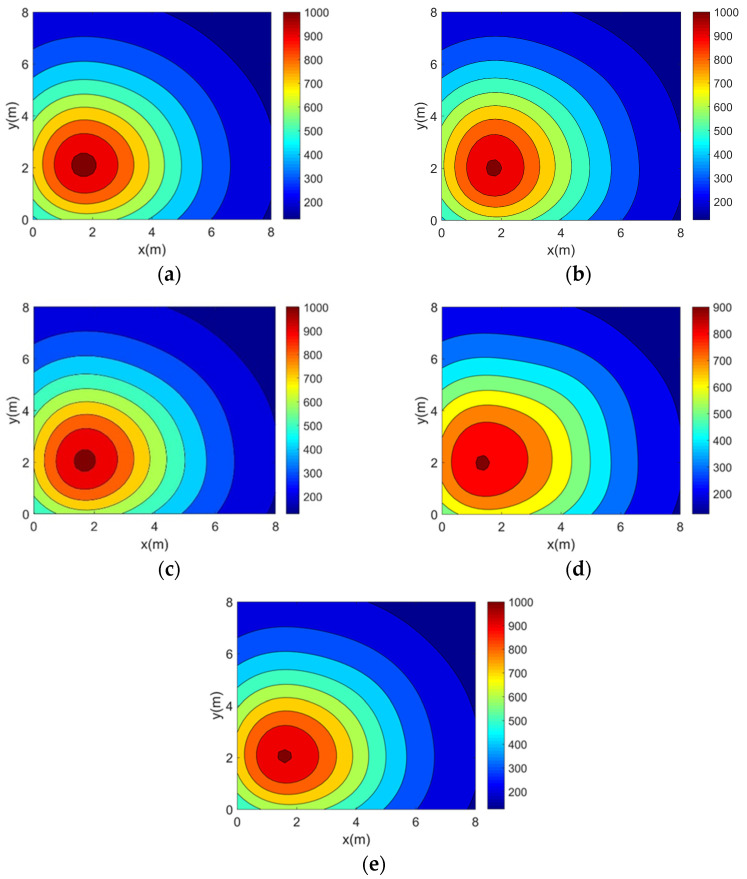
Single-peak temperature field reconstruction results. (**a**) Topology 1. (**b**) Topology 2. (**c**) Topology 3. (**d**) Topology 4. (**e**) Topology 5.

**Figure 7:**
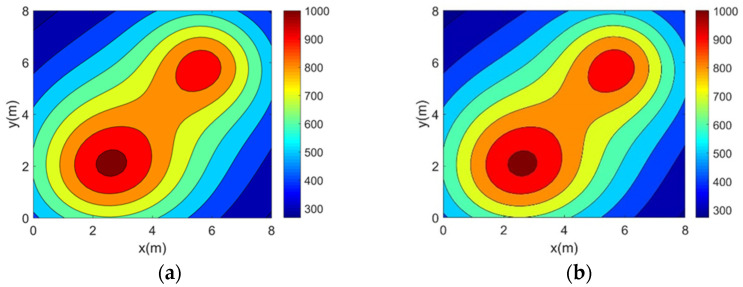

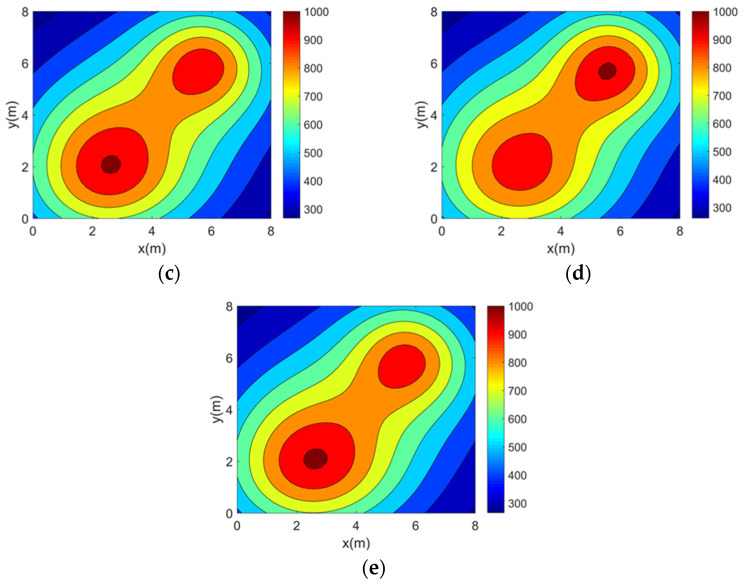
Double-peak temperature field reconstruction results. (**a**) Topology 1. (**b**) Topology 2. (**c**) Topology 3. (**d**) Topology 4. (**e**) Topology 5.

**Figure 8:**
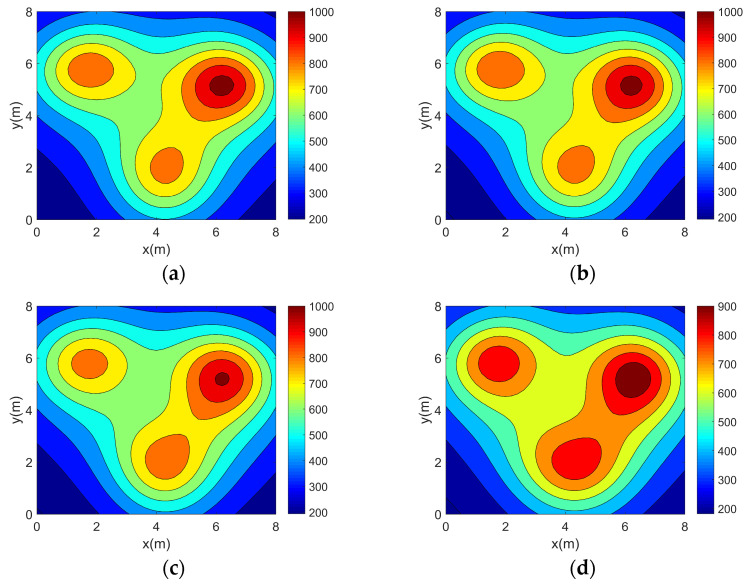

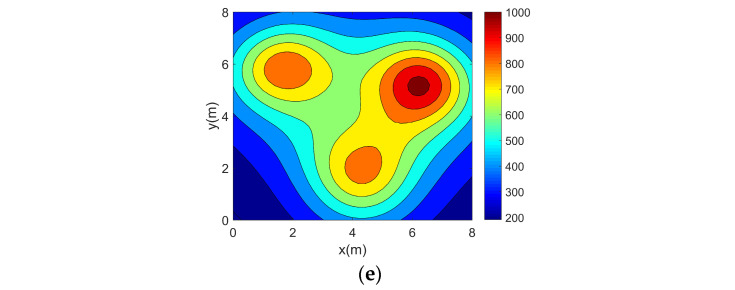
Three-peak temperature field reconstruction results. (**a**) Topology 1. (**b**) Topology 2. (**c**) Topology 3. (**d**) Topology 4. (**e**) Topology 5.

**Figure 9:**
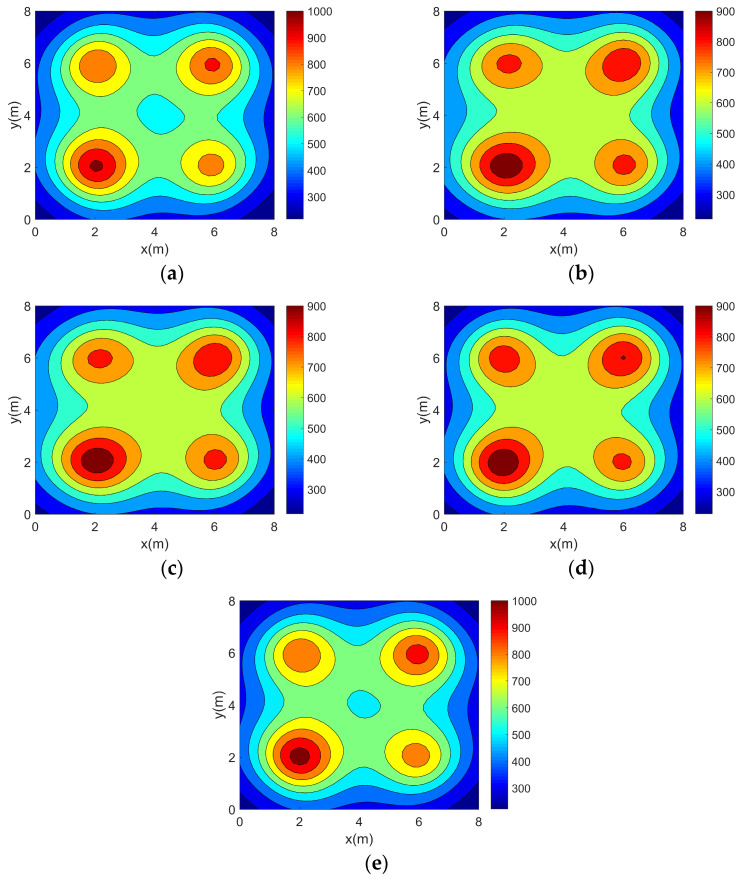
Four-peak temperature field reconstruction results. (**a**) Topology 1. (**b**) Topology 2. (**c**) Topology 3. (**d**) Topology 4. (**e**) Topology 5.

In this section, the accuracy experiment of the optimization method is carried out. Simulation reconstructions of four 2D asymmetric complex temperature fields using the five topologies mentioned above are carried out and the reconstruction results are shown in Figure 6, Figure 7, Figure 8 and Figure 9.

It can be seen from the above reconstructed images that all of the reconstruction results can better characterize the original distribution of the corresponding temperature fields. For the single-peak temperature field, all five topologies achieve good reconstruction results, though Topology 4 shows a larger deviation in temperature range compared with the original field. For the double-peak temperature field, Topology 4 accurately captures the number and location of heat sources but fails to describe the size relationship between the peaks. The other four topologies perform well with minimal differences. For the three-peak temperature field, the temperature range error of the reconstruction results of Topology 4 is relatively large, while the other topologies all have better reconstruction results. For the four-peak temperature field, Topology 2, Topology 3, and Topology 4 exhibit larger temperature range errors. Topology 3 and Topology 4, in particular, show lower resolution and fail to finely characterize the gradient in the center region. In contrast, Topology 1 and Topology 5 provide a more accurate description of the overall temperature distribution.

Table 7, Table 8 and Table 9 show the error data (obtained based on 41 × 41 = 1681 temperature calculation points) for the five topologies for the four temperature field reconstruction results.

Analyzing the above error data, it can be seen that all of the error indicators of the reconstruction results of Topology 5 are optimal. For Topology 5, the average $E_{remax}$ across the four temperature fields is 6.7953%, which is 2.6745%, 3.4971%, 2.8467%, and 3.06% lower than Topology 1, Topology 2, Topology 3, and Topology 4, respectively. The average $E_{remean}$ is 0.9963%, 0.2142%, 0.4778%, 0.7235%, and 1.2863% lower than the other four topologies, respectively. The $E_{rms}$ is 1.4587%, 0.2841%, 0.777%, 1.2926%, and 2.6582% lower than the other four topologies, respectively. It can be concluded that Topology 5 has higher reconstruction accuracy for all four temperature fields. This experiment shows that in asymmetric complex temperature fields, the topologies derived from the optimization approach in this study exhibit high accuracy.

B. Stability experiment of optimization results

In addition to accuracy, the measured topology must exhibit good stability (anti-interference capability) to ensure precise temperature field reconstruction in noisy environments. The stability validation experiment evaluates the five topologies by adding three levels of Gaussian noise (mean 0, standard deviations: 0.00005 s for low noise, 0.0001 s for moderate noise, and 0.0005 s for high noise) to the acoustic TOF. The expression for the acoustic TOF after the introduction of the noise is given as follows:

(28) $$t_{m}=t_{0}+t_{n}$$

where $t_{m}$ denotes the measured value of the TOF, $t_{0}$ denotes the true value of the TOF, and $t_{n}$ denotes the TOF measurement error caused by combustion noise.

The global reconstruction error data (obtained based on 41 × 41 = 1681 temperature calculation points) for the five topologies at different noise levels are shown in Table 10, Table 11, Table 12 and Table 13.

From the experimental results in the tables and for the identical temperature field, the reconstruction error increases with noise level. In various noise levels, Topology 5 is optimal in all error metrics and has the best stability for all temperature field reconstruction results. For the low noise, the $E_{rms}$ of single-peak, double-peak, three-peak and four-peak temperature fields reconstructed by Topology 5 are at least 1.7144%, 0.9276%, 2.0489% and 0.9372% lower than that of other topologies, respectively. For the moderate noise, the $E_{rms}$ of single-peak, double-peak, three-peak and four-peak temperature fields reconstructed by Topology 5 are at least 2.1946%, 1.0791%, 2.2026% and 1.9692% lower than that of other topologies, respectively. For the high noise, the $E_{rms}$ of single-peak, double-peak, three-peak and four-peak temperature fields reconstructed by Topology 5 are at least 10.4905%, 7.8515%, 19.5241% and 5.7876% lower than that of other topologies, respectively.

**Figure 10:**
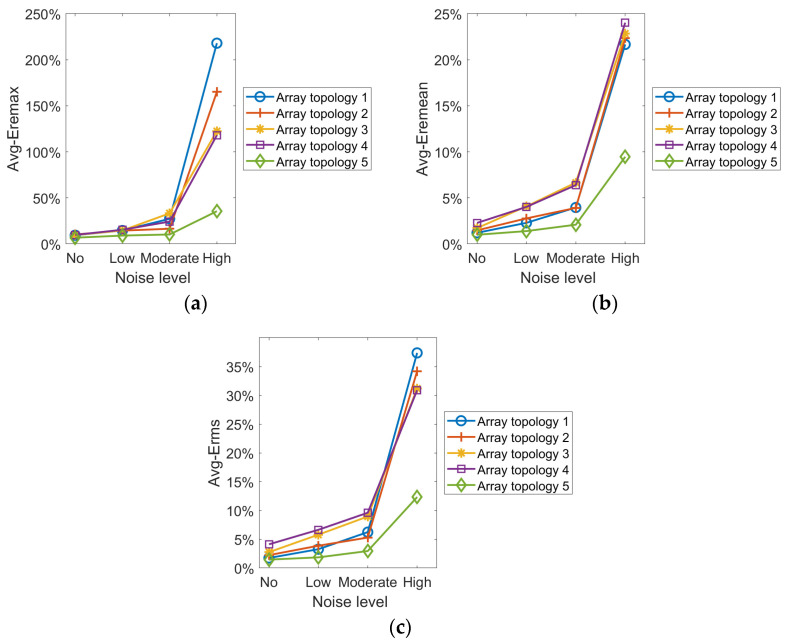
Curves of the mean value of the error at different levels of noise. (**a**) $E_{remax}$ (**b**) $E_{remean}$(**c**) $E_{rms}$.

To evaluate the overall performance of the topologies across the four temperature fields, the average reconstruction errors for each topology under different noise levels are calculated separately, as shown in Figure 10.

It can be seen from the preceding experimental data that the reconstruction error rises with noise level for the same field. Under the low noise condition, each topology can basically reconstruct all temperature fields. However, under moderate and high noise conditions, the reconstruction accuracy of all topologies, except for Topology 5, decrease significantly, and some fail to accurately depict the temperature field’s distribution. Different topologies vary in their ability to resist noise interference. Across noise levels, Topology 5 achieves overall optimization by synthesizing reconstruction results from each temperature field. The results demonstrate that the optimization strategy suggested in this work has a great anti-interference capacity in addition to having a high reconstruction accuracy.

## 4. Conclusions

Acoustic temperature measurement, as a non-contact temperature detection technique, has been developed and applied in several fields due to its unique advantages. This paper proposes a new adaptive optimization method for transducer topology in asymmetric complex temperature fields, building on previous research of the LQ-SVD algorithm and incorporating multiple sub-objectives. The method’s innovations include (1) optimization variables that ensure central symmetry, improving optimization efficiency and acoustic path coverage, and (2) optimization objectives that balance multiple factors to enhance reconstruction performance. The optimization objectives include acoustic path coverage, orthogonality, matrix condition number, and root mean square error of the reconstructed uniform temperature field. Weights are assigned to each sub-objective using the complex correlation method, balancing and optimizing multiple objectives to further improve reconstruction performance. Through the reconstruction experiments on four typical asymmetric complex temperature fields, the following conclusions can be drawn:

(1) Optimization objectives and optimization variables have an important impact on the reconstruction accuracy of temperature fields. Experimental results demonstrate a strong correlation between the proposed optimization objective and the reconstruction errors of all temperature fields, with correlation coefficients ranging from 0.4557 to 0.7672 for the root mean square error. The proposed optimization variable performs better across all temperature fields, with an average $E_{rms}$ of 1.4587%.
(2) The topology developed in this study demonstrates superior accuracy in asymmetric complex temperature fields, as confirmed by the accuracy assessment of the optimization results. Compared with the other four topologies, the average $E_{remax}$, $E_{remean}$, and $E_{rms}$ of the reconstruction results are reduced by at least 2.6745%, 0.2142%, and 0.2841%, respectively.
(3) The stability assessment of the topology optimization results shows that different optimization methods perform differently in resisting noise interference. The topology obtained by the optimization method in this paper has better stability in asymmetric complex temperature fields. Under three noise levels, the $E_{rms}$ of its reconstruction results increases by a maximum of 12.2903% and a minimum of 9.0037%.

The research presented in this paper demonstrates the feasibility and effectiveness of the proposed topology optimization method for acoustic temperature measurement, validated through simulations. While this paper focuses on square regions, its findings are also applicable to 2D non-square regions, offering new insights for acoustic temperature field reconstruction in 2D spaces. Future research will extend this optimization method to 3D scenarios, with a focus on constructing and studying multi-objective functions in 3D. Meanwhile, we will further promote the engineering application of the optimization method to verify the effectiveness and practicality of the present research results.

## Figures and Tables

**Table 1:** Correlation coefficients between different optimization objectives and the maximum relative error of the reconstructed temperature fields.

Objective	Single Peak	Double Peak	Three Peak	Four Peaks
1	0.0320	0.3312	0.0122	0.1029
2	0.0321	−0.0436	−0.0730	0.0326
3	0.3137	0.5119	0.5925	0.4330
**4**	**0.4173**	**0.6586**	**0.7136**	**0.5232**

**Table 2:** Correlation coefficients between different optimization objectives and the average relative error of the reconstructed temperature fields.

Objective	Single Peak	Double Peak	Three Peak	Four Peak
1	0.0968	0.3560	0.0122	0.1557
2	0.1060	−0.0114	−0.0495	−0.0492
3	0.3445	0.5739	0.6202	0.6535
**4**	**0.4033**	**0.6792**	**0.7101**	**0.7597**

**Table 3:** Correlation coefficients between different optimization objectives and the root mean square error of the reconstructed temperature fields.

Objective	Single Peak	Double Peak	Three Peak	Four Peak
1	0.1797	0.3634	0.0122	0.1516
2	0.1514	0.0002	−0.0456	−0.0329
3	0.3956	0.5761	0.6034	0.6354
**4**	**0.4557**	**0.6823**	**0.7057**	**0.7672**

**Table 4:** $E_{remax}$ of reconstructed temperature field with different optimization variables (%).

Variable	Single Peak	Double Peak	Three Peak	Four Peak	Average Value
1	0.1797	0.3634	0.0122	0.1516	7.8199
2	0.1514	0.0002	−0.0456	−0.0329	8.5252
**3**	**10.1191**	**3.2222**	**8.9920**	**4.8479**	**6.7953**

**Table 5:** $E_{remean}$ of reconstructed temperature field with different optimization variables (%).

Variable	Single Peak	Double Peaks	Three Peaks	Four Peaks	Average Value
1	1.2127	0.6489	2.0342	2.4354	1.5828
2	0.9232	0.5966	1.5003	1.2131	1.0583
**3**	**0.8910**	**0.5693**	**1.3386**	**1.1863**	**0.9963**

**Table 6:** $E_{rms}$ of reconstructed temperature field with different optimization variables (%).

Variable	Single Peak	Double Peak	Three Peak	Four Peak	Average Value
1	2.6050	0.8749	2.8836	3.3761	2.4349
2	2.1619	0.7320	1.8150	1.3475	1.5141
**3**	**1.9065**	**0.7103**	**1.7699**	**1.4482**	**1.4587**

**Table 7:** $E_{remax}$ of reconstructed temperature field for different topologies (%).

Topology	Single Peak	Double Peak	Three Peak	Four Peak	Average Value
1	13.3591	3.9851	13.1953	7.3395	9.4698
2	10.9287	5.5182	14.5353	10.1875	10.2924
3	13.1697	3.9229	10.5703	10.9051	9.6420
4	12.5463	5.4739	13.0838	8.3171	9.8553
**5**	**10.1191**	**3.2222**	**8.9920**	**4.8479**	**6.7953**

**Table 8:** $E_{remean}$ of reconstructed temperature field for different topologies (%).

Topology	Single Peak	Double Peak	Three Peak	Four Peak	Average Value
1	1.0230	0.6607	1.7146	1.4439	1.2105
2	1.0492	0.6694	1.7655	2.4124	1.4741
3	1.4126	0.7308	2.1177	2.6180	1.7198
4	2.3312	1.5674	2.6785	2.5534	2.2826
**5**	**0.8910**	**0.5693**	**1.3386**	**1.1863**	**0.9963**

**Table 9:** $E_{rms}$ of reconstructed temperature field for different topologies (%).

Topology	Single Peak	Double Peak	Three Peak	Four Peak	Average Value
1	2.4438	0.8192	1.9799	1.7281	1.7428
2	2.4092	0.8250	2.1854	3.5232	2.2357
3	3.1628	0.9728	3.0167	3.8527	2.7513
4	5.6979	2.6286	4.5234	3.6175	4.1169
**5**	**1.9065**	**0.7103**	**1.7699**	**1.4482**	**1.4587**

**Table 10:** Single-peak temperature field reconstruction errors at different noise levels.

Topology	$E_{remax}$			$E_{remean}$			$E_{rms}$		
	Low	Medium	High	Low	Medium	High	Low	Medium	High
1	19.4335	37.5037	333.999	1.7468	4.0232	27.5990	3.9229	10.3646	69.5368
2	11.0401	13.3797	265.4119	2.2016	3.7028	35.0836	4.0340	5.9104	63.1293
3	19.1751	30.2570	51.4074	4.3261	6.2025	15.1057	7.6060	8.4443	23.9015
4	20.4262	25.8747	56.8241	4.9687	7.0842	15.9226	10.4971	13.4270	24.2737
**5**	**11.7455**	**12.7366**	**31.7982**	**1.1384**	**1.9895**	**7.3393**	**2.2085**	**3.7158**	**13.4110**

**Table 11:** Two-peak temperature field reconstruction errors at different noise levels.

Topology	$E_{remax}$			$E_{remean}$			$E_{rms}$		
	Low	Medium	High	Low	Medium	High	Low	Medium	High
1	8.5519	20.8782	122.7031	1.9485	3.9488	18.0149	2.3939	5.0364	20.8521
2	7.3557	11.0989	64.2864	2.0507	2.8938	12.6346	2.3425	3.8040	20.9187
3	11.8877	23.4406	220.8364	3.0371	5.3590	29.2752	4.0037	7.2308	44.0717
4	11.8129	24.5661	102.6224	2.9946	6.1975	23.1693	3.6685	9.2776	27.1855
**5**	**5.8512**	**8.6872**	**39.8085**	**1.1534**	**2.2372**	**11.0489**	**1.4219**	**2.7249**	**13.0006**

**Table 12:** Three-peak temperature field reconstruction errors at different noise levels.

Topology	$E_{remax}$			$E_{remean}$			$E_{rms}$		
	Low	Medium	High	Low	Medium	High	Low	Medium	High
1	18.1153	37.7597	121.2096	3.2564	4.1947	22.3470	4.1013	4.9951	30.2977
2	18.0240	18.8321	277.1882	3.3322	4.6144	27.4961	4.4221	5.7196	34.8145
3	14.8481	58.5750	155.6020	4.3640	9.7767	30.9064	5.8622	13.0158	37.1940
4	14.4877	25.8231	205.7991	4.1827	6.1867	25.5675	6.5722	7.4752	33.0366
**5**	**11.4522**	**11.6918**	**44.1082**	**1.7983**	**1.9870**	**8.6353**	**2.0524**	**2.7925**	**10.7736**

**Table 13:** Four-peak temperature field reconstruction errors at different noise levels.

Topology	$E_{remax}$			$E_{remean}$			$E_{rms}$		
	Low	Medium	High	Low	Medium	High	Low	Medium	High
1	12.2916	13.1792	294.0211	2.2131	3.6593	18.6950	2.6537	4.5278	28.7892
2	21.5719	22.7303	53.5545	3.5173	4.4415	14.1202	4.6843	5.7226	17.8996
3	12.6955	20.8354	62.9527	4.5990	5.2985	16.0481	5.6806	7.0881	19.6315
4	14.9308	20.8368	106.9155	3.9443	6.0886	31.4322	5.8139	8.2330	39.0155
**5**	**7.2767**	**8.0469**	**26.5287**	**1.4967**	**2.1465**	**10.8240**	**1.7165**	**2.5586**	**12.1120**

## Data Availability

The raw data supporting the conclusions of this article will be made available by the authors on request.
